# Computational Reverse-Engineering of a Spider-Venom Derived Peptide Active Against *Plasmodium falciparum* SUB1

**DOI:** 10.1371/journal.pone.0021812

**Published:** 2011-07-27

**Authors:** Giacomo Bastianelli, Anthony Bouillon, Christophe Nguyen, Elodie Crublet, Stéphane Pêtres, Olivier Gorgette, Dung Le-Nguyen, Jean-Christophe Barale, Michael Nilges

**Affiliations:** 1 Unité de Bioinformatique Structurale, Département de Biologie Structurale et Chimie, Paris, France; 2 CNRS, URA2185, Paris, France; 3 Unité d'Immunologie Moleculaires des Parasites, Département de Parasitologie et de Mycologie, Paris, France; 4 CNRS, URA2581, Paris, France; 5 SYSDIAG, CNRS UMR3145 CNRS-BioRad, Montpellier, France; 6 Institut Pasteur, Plate-Forme 5 - Production de Protéines Recombinantes et d'Anticorps, Paris, France; National Institute for Medical Research, Medical Research Council, London, United Kingdom

## Abstract

**Background:**

Psalmopeotoxin I (PcFK1), a protein of 33 aminoacids derived from the venom of the spider *Psalmopoeus Cambridgei*, is able to inhibit the growth of Plasmodium falciparum malaria parasites with an IC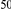 in the low micromolar range. PcFK1 was proposed to act as an ion channel inhibitor, although experimental validation of this mechanism is lacking. The surface loops of PcFK1 have some sequence similarity with the parasite protein sequences cleaved by PfSUB1, a subtilisin-like protease essential for egress of *Plasmodium falciparum* merozoites and invasion into erythrocytes. As PfSUB1 has emerged as an interesting drug target, we explored the hypothesis that PcFK1 targeted PfSUB1 enzymatic activity.

**Findings:**

Molecular modeling and docking calculations showed that one loop could interact with the binding site of PfSUB1. The calculated free energy of binding averaged −5.01 kcal/mol, corresponding to a predicted low-medium micromolar constant of inhibition. PcFK1 inhibited the enzymatic activity of the recombinant PfSUB1 enzyme and the in vitro *P.falciparum* culture in a range compatible with our bioinformatics analysis. Using contact analysis and free energy decomposition we propose that residues A14 and Q15 are important in the interaction with PfSUB1.

**Conclusions:**

Our computational reverse engineering supported the hypothesis that PcFK1 targeted PfSUB1, and this was confirmed by experimental evidence showing that PcFK1 inhibits PfSUB1 enzymatic activity. This outlines the usefulness of advanced bioinformatics tools to predict the function of a protein structure. The structural features of PcFK1 represent an interesting protein scaffold for future protein engineering.

## Introduction

Despite recent scaling up of control efforts, malaria remains a major public health problem [1]. The development of novel control tools is urgently needed as *Plasmodium falciparum* has become resistant to multiple drugs [2] while vector mosquitoes resist to insecticides in many areas. The development of novel antimalarials is a priority [3]. Recently, parasite proteases involved in egress and/or invasion of the host erythrocytes have emerged as potential drug targets. In particular, the subtilisin-like serine protease PfSUB1 is involved in the maturation of parasite proteins implicated in the egress of the *P.falciparum* merozoite from the infected erythrocyte [4] and in the maturation of the major merozoite surface protein (MSP1) required for successful erythrocyte invasion [5].

In addition to therapies based on small molecules such as chloroquine, artemisinins, quinine and atovaquone [6], the development of new classes of molecules based on proteins or peptidomimetics [7] [8] is an active field of research. Among antiplasmodial bioactive proteins, dermaseptin S4 (DS4) is able to inhibit irreversibly the growth of the parasite, through a cytotoxic hemolytic activity [9]. Dermaseptin S3, a related protein, acts in an analogous manner to DS4 by inhibiting *P. falciparum* growth *in vitro*, but without hemolytic activity [10]. Choi *et al.* have isolated from the venom of the tarantula *Psalmopoeus cambridgei* two novel peptides that inhibit the intra-erythrocytic cycle of *P. falciparum* [11]. Psalmopeotoxin I (PcFK1) is composed of 33 residues, while Psalmopeotoxin II (PcFK2) has 28 residues; both have three disulfide bridges and belong to the cystine knot motif superfamily. These two peptides inhibit the growth of the parasite with an IC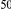 in the low micromolar range, show no antifungal or antibacterial activity and are neither hemolytic nor do they affect the growth or viability of human epithelial cells. The authors concluded that PcFK1 and PcFK2 interact specifically with *P. falciparum* parasitized erythrocytes. Later on, the NMR structure of PcFK1 revealed that it belongs to the ICK structural superfamily with structural determinants common to several neurotoxins that act as ion channel effectors. Based on this structural similarity it was proposed that the molecular target for PcFK1 could be an ion channel [12], but no experimental data was provided to support this hypothesis. Using bioinformatics analysis, protein-protein docking methods and free energy calculations we formulate here the hypothesis that PfSUB1 is a target for this small protein. We confirm this hypothesis by experimental testing on PfSUB1 specific enzymatic assay using a PfSUB1 purified and active recombinant enzyme. The understanding of how PcFK1 may interact with PfSUB1 provides important information for using this small protein as a scaffold in order to improve its inhibitory activity using computational protein design [13].

## Results

### Sequence Comparison

**Figure 1:**
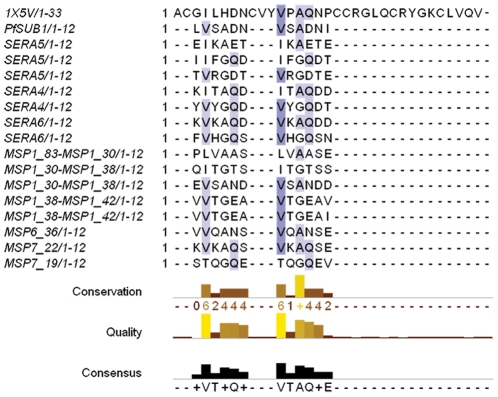
Alignment of PcFK1 with substrate sequences of PfSUB1. The sequence alignment of PcFK1 (pdb: 1X5V) with the sequences recognized by PfSUB1 shows a comparable residue profile for Site 1 and Site 2. In particular, Site 2 shows the highest sequence similarity with the most conserved residues among the substrate sequences. The shades of blue indicate the degree of conservation among the sequences. The SERA4 and SERA6 processing sites are predicted from sequence alignments and homology with the experimentally determined SERA5 processing sites [4]. All other sites shown here were experimentally determined by amino-acid sequence analysis [5].

The sequence of the observed or predicted PfSUB1 cleavage site in its various protein substrates is listed in Figure 1. Like other subtilisin-like proteases, PfSUB1 recognizes a broad range of sequences, but shows a pattern of preferred amino acids with hydrophobic aliphatic side chains in P4 while polar or positively charged residues are often present in P3. Small amino acids (gly, ala) are preferred in P2 while for all other positions (P1, P1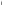, P2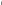, P3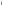), the enzyme prefers polar and negatively charged side-chains.

As shown in Figure 1, two regions from PcFK1, called here Site1 and Site 2, show a profile comparable to the PfSUB1 substrate sequences. Both display residues present in the natural substrate sequence(s). For Site 1, the greatest similarity is localized at P1 and P1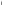, with the aspartate and asparagine residues, respectively, being identical to the autocatalytic sequence of PfSUB1. The P4 presents an isoleucine that is fairly similar to valine, the most represented residue in P4. Site 2 shares even more similarities than Site 1. Position P4, P2 and P1 are occupied by a valine, an alanine and a glutamine respectively, the most represented residues among all substrate sequences. These observations led us to formulate the hypothesis that PfSUB1 could be a target for the PcFK1 parasite inhibitor.

### Structural Analysis

**Figure 2:**
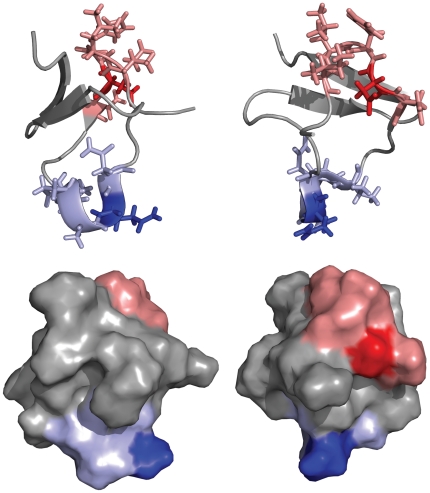
Structure sites localization. Two views of the structure of PcFK1 (pdb: 15XV). (top) Site 1 is highlighted in red sticks while Site 2 in blue sticks and residues in position P1 are shown in darker blue or red. (bottom) Both sites are at the protein surface and therefore accessible to protein-protein interaction.

**Figure 3:**
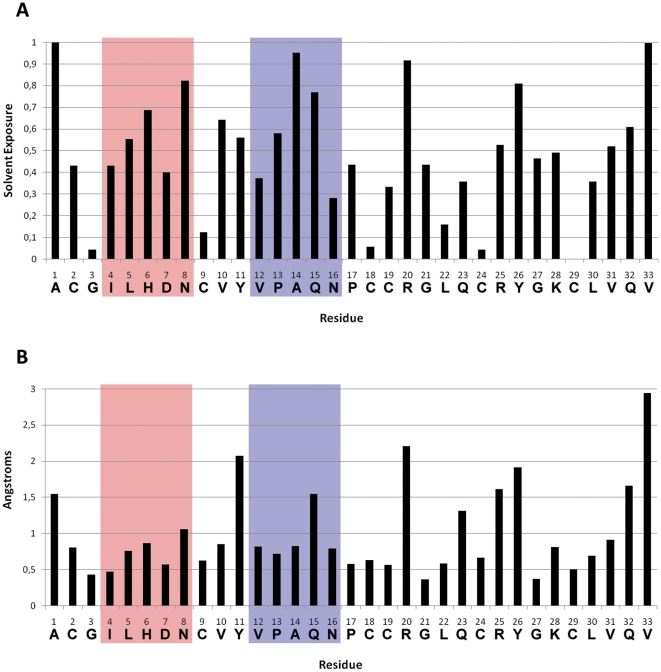
Solvent exposure and RMSF for PcFK1. The two sites are highlighted in red for Site 1 and blue for Site 2 where D7 and Q15 are the P1 position of Site 1 and Site 2, respectively. (A) shows that most of residues are solvent accessible apart from the cysteine side-chains forming disulphide bridges. The glutamine in position 15 is significantly more exposed than the aspartate in position 7. The RMSF analysis (B) shows that PcFK1 has a fairly rigid structure with few exceptions at the N and C-terminal, and the tyrosines and arginines. The P1 residue of Site 2 (Q15) fluctuates more than its counterpart of Site 1 (D7).

The sequence similarities indicate two possible sites that could interact with PfSUB1. As Figure 2 shows, these sequences are on the surface and accessible for a potential interaction with a protein partner. An analysis of the solvent accessible surface with DSSP on the 25 NMR conformers (Figure 3.A) showed that both sites are quite solvent accessible, but that Site 2 shows a higher solvent accessibility of position P1 (Q15) compared to the P1 of site 1 (D7). Position P1 in protein inhibitors has a central role in the interaction with serine-proteases [14]. In PfSUB1, the importance of the P1 was investigated using recombinant PfSUB1 with a mutation in the P1 position of the autocatalytic cleavage sequence. A substitution with a leucine in this crucial position resulted in a partially maturation-defective enzyme that was converted to active protease with much reduced efficiency, suggesting a central role for the P1 residue of the autocatalytic cleavage sequence [15]. We also analyzed the fluctuation from the average structure of each residue, finding that Site 2 is more flexible, in particular position Q15 (Figure 3.B), the potential P1 position. A relatively flexible P1 will allow its side-chain to adapt to the S1 pocket more efficiently. Overall, these observations suggest that Site 2 could be more likely responsible for an interaction with PfSUB1.

### PfSUB1 Modeling

The validation of a model is a critical step in comparative homology modeling. After creating a pool of models using the homology modeling suite Modeller (see Material and Methods), we selected models with the most precise catalytic site geometry and an overall good structure quality. Correct distances among key atoms in the catalytic site are necessary for the function of the enzyme and can be used as indicator of a reliable model. We analyzed the geometry for all templates we used in our homology modeling protocol and we defined a range for each distance. A model is accepted for further analysis only if all its distances fall within the range that we defined (within lower and upper range, see Table 1).

**Table 1:** Subtilisin catalytic site geometries.

Distance	lower-range	upper-range
HIS@CA-SER@CA	8.3	8.72
HIS@CB-SER@CB	6.44	6.89
HIS@CA-ASP@CA	7.22	7.46
HIS@CB-ASP@CB	5.83	6.61
SER@CA-ASP@CA	9.87	10.11
SER@CB-ASP@CB	8.15	8.53
SER@OG-HIS@NE2	2.57	3.36
ASP@CG-HIS@ND1	3.15	3.35
ASN@CG-SER@CB	6.34	6.74
SER@OH-surface	1.58	1.60

The distances were obtained by analyzing the geometry of the catalytic site (Asp-His-Ser) of the templates used in the modelling of PfSUB1. The Asn is the residue forming the oxyanion hole.

**Figure 4:**
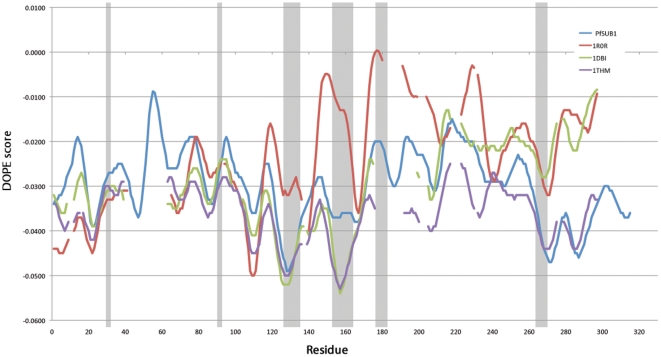
DOPE profile comparison with templates. The graph shows the DOPE by-residue profile of our best model of PfSUB1 against three templates used in the modeling where in gray are shown the residues forming the binding region. Despite a region with a high energy (around residue 60) all regions forming the binding region show a relatively good DOPE energy. Missing values in the templates are due to gaps in the sequence alignment.

**Figure 5:**
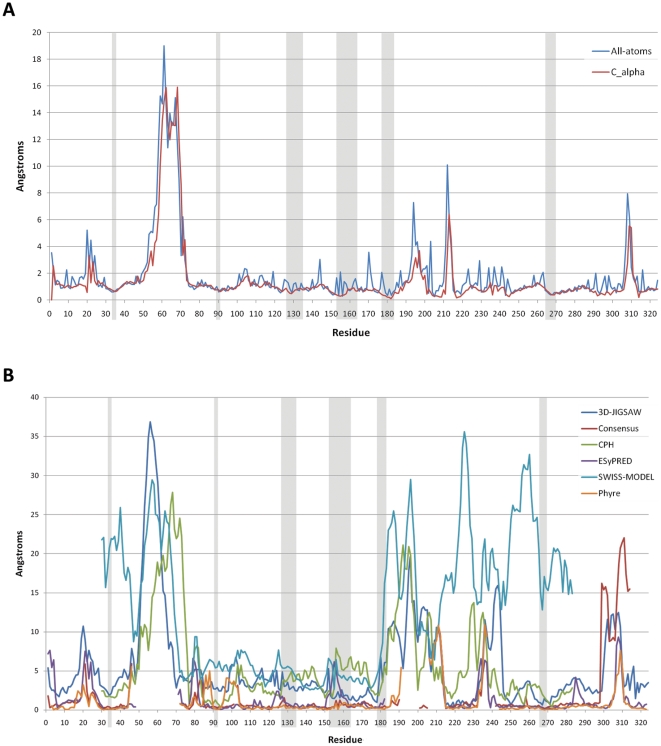
RMSD among models of PfSUB1. In A the RMS fluctuation on a per-residue basis is plotted for the 10 best models. Gray rectangles highlight the residues forming the binding region. (B) Comparison of our best model with models obtained from several modelling servers. Profit was used to calculate by-residue RMSD where the structure alignment was performed using Needleman and Wunsch sequence alignment.

We ranked the filtered models according to quality using several structure analysis programs included in the online server SAVS (see Material and Methods), selecting the best 10 for further analysis. Of the 10 models we selected the one that ranked best of DOPE (Discrete Optimized Protein Energy) Z-score [16] (−0.97) and Prosa Z-score (−5.98). In Table 2 we show the comparison of the DOPE score and DOPE Z-score of our best model to the templates used in the modeling procedure. The negative value close to −1.0 suggest that the model is of good quality and likely to be native-like. We also plotted the DOPE energy by-reside as shown in Figure 4. Despite a region around residue number 60 with high energy, all residues forming the binding regions have a good DOPE energy if compared to the templates. To further evaluate the quality of our modeling procedure, we compared the models among each other and also with models obtained by several homology modeling servers. In Figure 5 we plotted the RMSD from the average structure of our 10 best models on a per-residue basis. Highly fluctuating regions can be either flexible loops or regions incorrectly modeled because of missing structural information in the templates. Considering the overall rigidity of subtilisins and of serine proteases [17], the regions with high flexibility are probably not correctly modeled. The results showed that the residues that constitute the binding region are structurally conserved and that regions with high RMSD are not close to the binding region. By comparing our best model with models obtained by several homology modeling servers (Figure 5.B) we observed that for the residues forming the binding interface our models have the lowest RMSD. This increases the confidence that these positions are probably correctly modeled.

**Table 2:** Comparison of PfSUB1 model to templates.

Structure	DOPE score	DOPE Z-score
PfSUB1	−35609.20	−0.97
1BH6	−31888.00	−2.197
1DBI	−32528.70	−2.168
1GCI	−30067.13	−2.266
1R0R	−31499.16	−2.062
1SCJ	−31205.20	−2.046
1THM	−31950.16	−2.269
1TO2	−31323.86	−1.914

The model of PfSUB1 shows a negative DOPE Z-score, usually an indication of a model of good quality. Scores lower or around −1.0 are likely to be native-like. The other templates show lower Z-score because they are experimental X-ray structures.

Table 3 shows the overall RMSD of each server model to our PfSUB1 model and their coverage. Models obtained from Phyre and ESyPred3D servers are the most similar to ours with RMSD of 3.78 Å and 3.66 Å, respectively. The analysis of the trajectories with molecular dynamics simulations revealed that the model is stable and behaves similarly to two subtilisins used as templates (data not shown).

**Table 3:** RMSD to server models.

Web-server	RMSD	Interface RMSD	Coverage %
3D-JIGSAW [53]	6.63 Å	2.14 Å	100%
Consensus Server [54]	5.41 Å	2.03 Å	82%
CPH [55]	5.4 Å	1.89 Å	78%
ESyPred3D [56]	3.66 Å	1.65 Å	85%
SWISS-MODEL [57]	5.24 Å	2.78 Å	78%
Phyre [58]	3.78 Å	1.49 Å	85%

PfSUB1 models were built using several available homology modeling servers. The models obtained with ESyPred3D and Phyre servers are the structurally closest to our model of PfSUB1 with respectively a RMSD of 3.66 Å and 3.78 Å. The interface RMSD were calculated on the residues (see Materials and Methods) that defines the binding pocket. The coverage refers to the percentage of our model structure that was covered by other servers.

### Docking to PfSUB1

**Figure 6:**
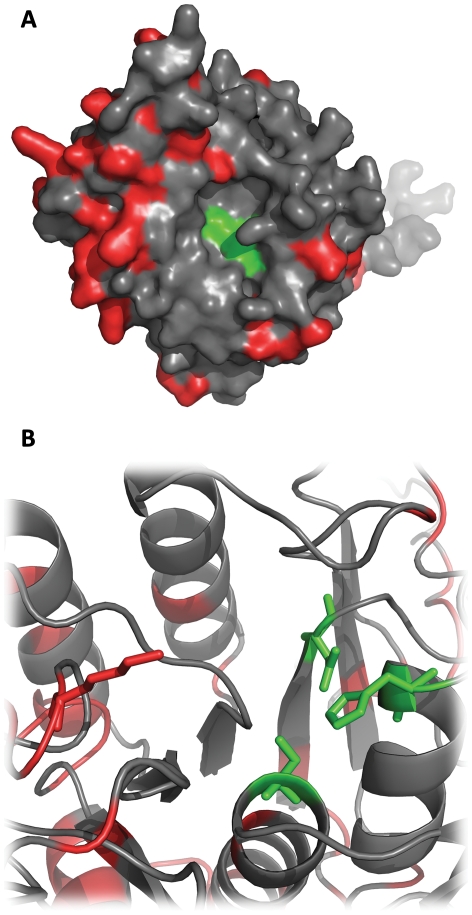
Docking Results. The plot illustrates the 2000 solutions with the highest Z-Score obtained from the ensemble docking performed employing the 25 NMR conformers of PcFK1 against the model of PfSUB1. The results show that Site 2 has a distance of the carbon-carbonyl to the hydroxyl of the catalytic serine close to the average value observed in many other complexes (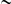
2.7 Å). This suggests that Site 2 can be the region responsible for the interaction with PfSUB1.

We employed an ensemble–docking strategy, using 25 NMR conformers of PcFK1, and used the distance between the carbonyl-carbon of P1 and the hydroxyl group of the catalytic serine (S268) as a criterion for a well-docked model. Analyzing some X-ray structures of complexes of subtilisins with their protein inhibitors we observed an average distance of 2.72 Å. The docking results shown in Figure 6 indicate that Site 2 has a distance that is closer to 2.72 Å when compared to Site 1, confirming that Site 2 is more likely the region responsible for the inhibition of PfSUB1. Nonetheless, all docking solutions show a larger distance than what is observed in the X-ray structures of complexes. This can be explained by the imperfections in the model and a docking protocol that does not include enough flexibility, in particular at the level of the receptor (PfSUB1).

### Refinement of docked structures

**Figure 7:**
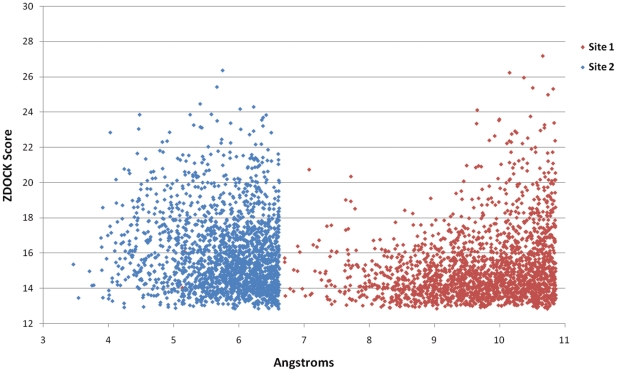
RMSD Map. The plot shows the distribution of the RMSD for the best 10 docked solutions before and after refinement where the average structure was set at (0,0). The map was obtained by calculating the RMSD distance matrix among all solutions and their averages before and after the refinement. The RMSD distance matrix was then represented in a 2 dimension plot. While starting docked solutions are dispersed with RMSD values even >6 Å, after the refinement the RMSD is <3 Å, indicating a convergence to a more similar docked conformation.

We therefore took the best 10 solutions based on the distance criterion and refined them using a restrained molecular dynamics protocol where we restrained the distance between the hydroxyl group of the catalytic serine and the carbonyl-carbon of Gln in P1. In a related study, we applied restrained molecular dynamics in order to refine docking poses obtained from an ensemble-docking protocol, showing the importance of this refinement in order to obtain high-accuracy docking models (Bastianelli *et al.*, submitted). In Table 4 we compared the MDs of the ten solutions with and without refinement. The change of RMSD is in all cases less than .5 Å suggesting that the refinement procedure do not introduce distortion in the structure. In addition, we evaluated if the refinement procedure could modify the structure of the spider toxin by comparing the NMR ensemble with the structures after refinement. In the NMR ensemble, the average RMSD from the average structure is 1.62 Å . We ran MDs (on the 5 most representatives structures determined by clustering) and we found an average deviation from the initial structure in the first 1.720 ns of 1.32 Å . We then calculated the RMSD for the spider toxin structure during the ten refinements and compared to the initial structure of the complex. In all ten cases the deviation was less than 2 Å suggesting that the structures are also not distorted. After the refinement, the structures of the complex show a reduction of RMSD to their average structure indicating that the refinement procedure allows different docking poses to converge to a more similar docked conformation (Figure 7). We also calculated the free energy of binding on the 10 complexes we refined. Free energy calculations based on MM-GBSA and MM-PBSA have been used in the past to estimate successfully absolute free energy of bindings [18] [19] [20] [21]. The average free energy of binding calculated from the MDs of the 10 complexes was −5.01
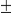
6.27 kcal/mol, which corresponds to an inhibition in the low to medium micromolar range.

**Table 4:** Comparison of RMSD along MDs in refined and non-refined docked complexes.

Solution	RMSD NO-REF	RMSD REF
1	2.62	2.38
2	2.67	2.41
3	2.69	2.36
4	3.01	2.89
5	2.99	2.76
6	2.65	2.33
7	2.77	2.40
8	2.62	2.32
9	2.98	2.53
10	2.78	2.43

The RMSD from the first snapshot of the MDs were calculated for the 10 docked solutions with and without refinement. In all cases the difference in terms of RMSD is less than .5 Å meaning that the refinement procedure does not introduce distortions in the structures. For both refined and non-refined MDs we calculated the RMSD over the 1 ns unrestrained MDs. NO-REF: solutions not refined. REF: solutions refined.

### Enzymatic and Culture Tests

**Figure 8:**
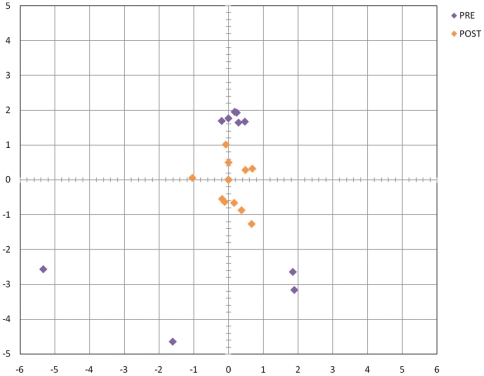
PfSUB1 and PvSUB1 structural comparison. Both figures show the model of PfSUB1 and the distribution of sequence differences between PfSUB1 and PvSUB1 (in red). The residues constituting the catalytic triad (Asp,His,Ser) are represented in green in A and as green sticks in B. In Figure B we highlight lysine 203 (red sticks) that is replaced by an arginine in PvSUB1. This is the only sequence difference that we can find in the vicinity of the binding pocket.

**Figure 9:**
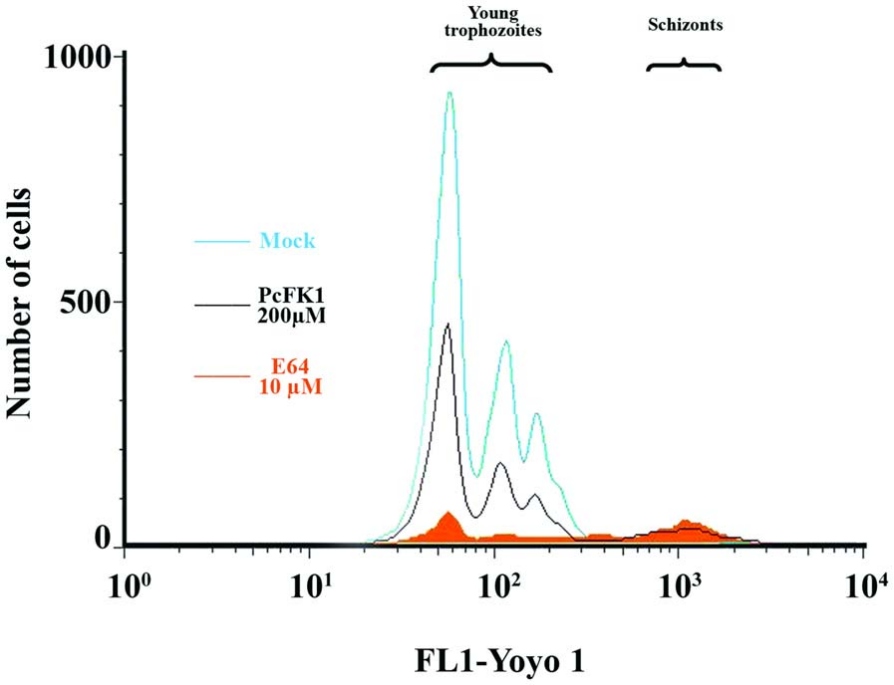
Analysis of merozoites egress/invasion steps on *P. falciparum in vitro* culture. In vitro synchronized culture of P. falciparum composed of 0.5% of segmented schizonts was incubated for 12 hr in serum-containing medium and schizonts transition to newly formed trophozoites was analyzed by flow cytometry. Gates were converted to two-dimensional plots illustrating the expected merozoites egress defect in presence of 10 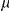
M E64 [51], but also in presence of 200 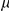
M of PcFK1. 91.6% and 56.2% of inhibition of newly formed trophozoites and equivalent segmented schizont accumulation were observed with 10 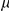
M E64 and 200 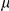
M PcFK1 respectively, when compared to the mock control. Mock condition corresponds to a classical *P. falciparum* culture in presence of 2% DMSO, the vehicle of PcFK1.

We then tested the capacity of PcFK1 to inhibit the PfSUB1 enzyme activity in an enzymatic assay using the recombinant PfSUB1 enzyme and a peptidic substrate derived from MSP1. PcFK1 indeed inhibited the enzyme with a constant of inhibition (Ki) of 29.3 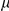
M
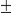
10.6 (SEM), confirming an inhibitory activity of PcFK1 in the medium micromolar range. If we translate this Ki value into free energy of binding we obtain −6.37 Kcal/mol, a value that deviates by less than 2 kcal/mol from our predictions of −5.01
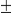
6.27 kcal/mol. To further document PcFK1-mediated inhibitory capacity, we also tested PcFK1 against the recombinant Plasmodium vivax SUB1 (PvSUB1). PfSUB1 and PvSUB1 share more than 68% sequence identity of their catalytic regions, most of sequence differences being located outside the binding pocket (Figure 8). Due to this high degree of sequence similarity they cleave substrates with comparable sequences. PcFK1 inhibits PvSUB1 with a Ki of 36.3 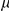
M
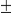
6.9 (SEM). We determined the EC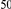 of PcFK1 on the asynchronous culture of *P. falciparum* intra-erythrocytic cycle and found a high micromolar inhibition of 116 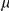
M
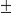
6 (SEM). PfSUB1 is involved in both the merozoite egress from red-blood cells (RBC) and their subsequent invasion into fresh RBC. The results on Figure 9 show that PcFK1 indeed inhibits the egress-invasion steps of *P. falciparum* merozoites (Figure 9.D). The experiments performed on the PfSUB1 and PvSUB1 active recombinant enzyme and on the *P. falciparum* culture *in vitro* further confirm our initial hypothesis that PcFK1 indeed targets the parasite SUB1 enzyme activity.

### Modeling the interaction

**Figure 10:**
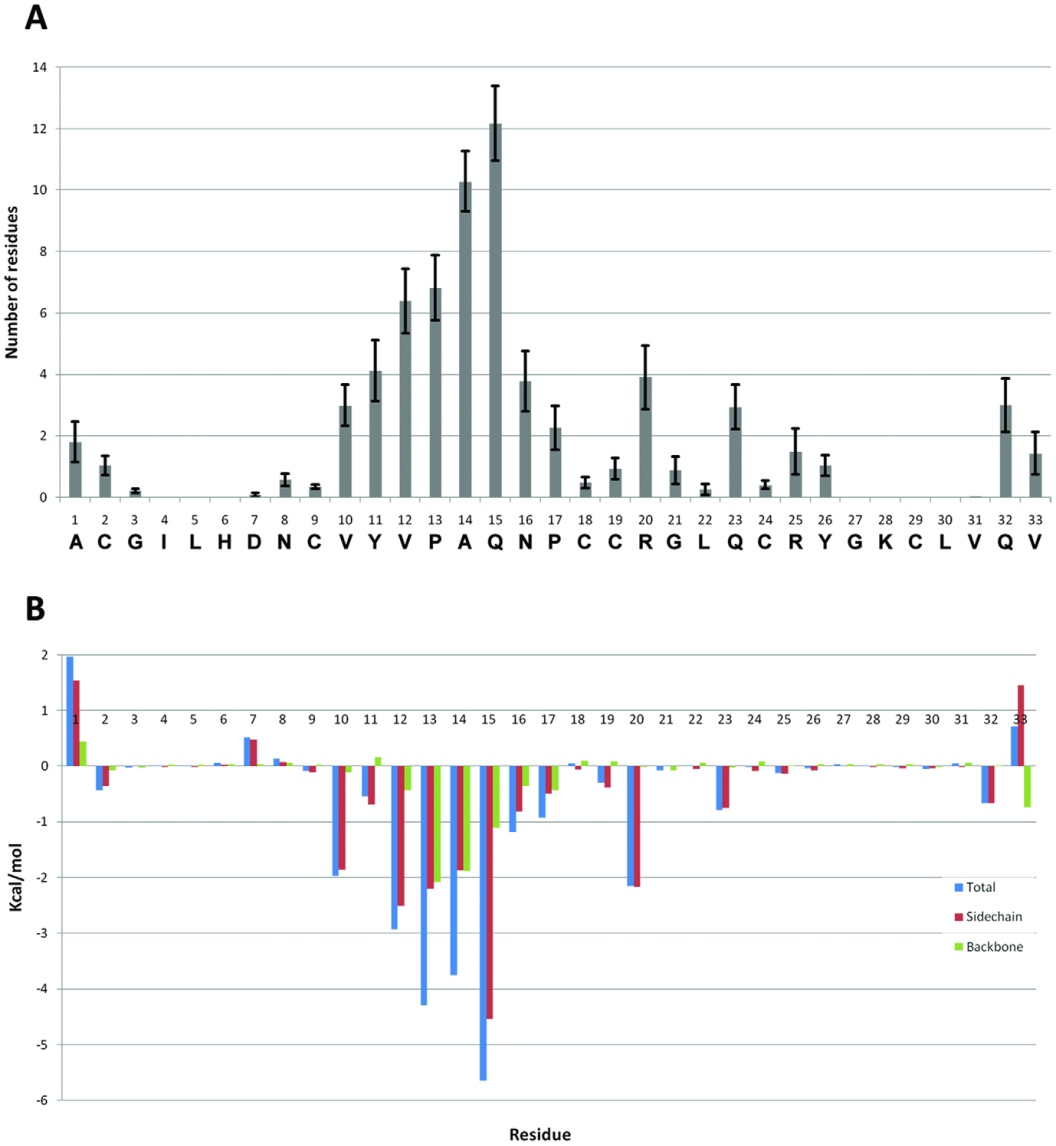
Interacting residues. (A) Number of residues of PfSUB1 interacting with each residue of PcFK1 on average from the 10 MDs of the best solutions. Most of contacts are made by residues in P2 (A14) and P1 (Q15). (B) Free energy decomposition on a per-residue basis with separate contribution of side-chain and backbone.

Being able to identify the mode of interaction of PcFK1 is crucial when envisioning a protein engineering approach to improve PcFK1 biological activity. The first step is the identification of the residues that are important for the interaction of PcFK1 with PfSUB1. The neighbor search in Figure 10 shows the number of average residues of PfSUB1 that are in contact with each of PcFK1 residue in all 10 MD trajectories. Residues in P2 and P1 show the highest number of contacts with PfSUB1 residues, of 10.3 and 12.2, respectively. Overall, Site 2 makes more residue contacts than other regions of PcFK1. A more informative by-residue decomposition of the relative free energy (not decomposing the entropy) shows that the P1 residue contributes for −5.65 Kcal/mol, accounting for 25% and P2 for a 17% of the total free energy of binding (Figure 10). More than 75% of the free energy of binding from PcFK1 comes from Site 2 residues (P4, P3, P2, P1, P1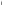). Position P1 and P2 seem to be the most important residues for the interaction with PfSUB1, and they are the most interesting residues for being engineered.

## Discussion

PcFK1 was previously shown to inhibit the growth of *P. falciparum* at the erythrocytic stage but no molecular target was assigned. Based on a series of observations that included sequence analysis, docking to the model of the enzyme and free energy calculations, we hypothesized in this paper that PcFK1 could act as an inhibitor of PfSUB1. The enzymatic assays not only showed that indeed PcFK1 inhibits the recombinant purified active PfSUB1 enzyme, but also with an affinity (K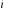) in very good agreement with our bioinformatics calculations. PcFK1 inhibits not only PfSUB1 but also PvSUB1, its orthologous protein in *Plasmodium vivax*, the main causative agent of malaria outside the african continent. This was an important additional confirmation of our findings on PfSUB1 since the two enzymes are likely to have the same biological function and display very close structures of the binding region (Figure 8). The inhibition of both *P. vivax* and *P. falciparum* by PcFK1 indicates that the interaction is mediated by a sequence in PcFK1 that is similar to the substrate sequences recognized by SUB1. Our computational analysis showed that Site 2 is most likely the sequence responsible of this inhibition. The tests performed on the *in vitro* culture of *P. falciparum* intra-erythrocytic cycle further confirmed the enzymatic results indicating that the anti-*Plasmodium* activity of PcFK1 is mostly directed towards the daughter parasite egress from and subsequent invasion into the human erythrocytes, the biological stages where PfSUB1 is known to play a crucial role. It is noteworthy that the pro-peptide of PfSUB1 is also a strong inhibitor of the enzyme [22] but due to its large size (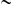
30 kDa) and since its anti-parasitic effect on the *P. falciparum in vitro* culture has not been established, it is not an interesting scaffold for engineering a potent anti-parasite peptidic inhibitor. By contrast, PcFK1 is small, rigid and it belongs to the family of proteins with a cysteine knot motif. Proteins with this unique structural scaffold can be extraordinarily stable and have a broad range of bioactivities. This can be exploited for developing more active peptides, via molecular engineering approaches [23].

The docking analysis and the MD based refinement proposed a model of interaction where residues in P1 (glutamine) and P2 (alanine) establish most contacts with PfSUB1. The understanding of the structure-function relationship for PcFK1 allows to gain more insights on the structure of SUB1 and to guide the rational engineering of PcFK1 in order to improve its inhibitory activity. Most of protein engineering studies [24] use some of the methods we described in this study with the objective of engineering a protein scaffold for a specific biological activity, such as binding to another protein target [25]. In this paper we used these methods in a predictive fashion, in a computational reverse engineering process. Our results show that such a strategy could indeed be powerful to assign a molecular target to peptidic compounds, which are known to display a biological effect, but remain orphan of biological target. Approaches similar to the one we described in this study will have an increasing importance as our understanding of protein-protein and protein-ligand interactions advances. Molecular modeling methods for docking proteins [26] and calculate free energy [27] have been used with some successful results in computational protein engineering studies and advancements in algorithms and computational power will provide a greater help in the future to guide protein engineering. We can envision using similar methods to the ones we used in this paper to engineer PcFK1 in order to improve its inhibitory activity against PfSUB1. This could lead to new protein-based therapeutic approaches targeting novel mechanisms of action.

## Materials and Methods

### Bioinformatics analysis

#### Protein modeling

To obtain a homology model of PfSUB1 we retrieved sequences from the Uniprot database [28] (www.uniprot.org) and structure files of the templates from the PDB [29] (www.rcsb.org). The following templates were used: 1TO2, 1THM, 1SCJ, 1R0R, 1GCI, 1DBI, 1BH6. Template structures were cleaned of any heteroatom or water molecule and sequences were submitted to the Expresso server [30] (3D-TCOFFEE) using default parameters. 200 models were generated with Modeller v9.1 [16] using slow molecular dynamic refinement and repeated optimization.

Profit (http://www.bioinf.org.uk/software/profitt/index.html) was used to run structure alignment and calculate RMSD using the ITERATE option. Considering the high sequence identity (>68%) shared between PvSUB1 and PfSUB1 active site domains, we modelled PvSUB1 starting from our model of PfSUB1. Binding pocket residues were defined according to the interaction of canonical inhibitors with subtilisins. PfSUB1: 152–162, 127–134, 266–269, 179–182, 90 (His), 34 (Asp), 268 (Ser); PvSUB1: PvSUB1: 152–162, 127–134, 265–268, 179–182, 90 (His), 34 (Asp), 267 (Ser). Structure quality was assessed with several softwares such as Procheck [31], WhatCheck [32] [33], Errat [34] and Verify3D [35] [36] using SAVS server (nihserver.mbi.ucla.edu/SAVS1/). Prosa calculations were obtained using ProSA-web [37].

The NMR structure of PcFK1 (PDB: 1X5V) was retrieved from the PDB [29]. The RMSF was calculated with AMBER [38] using the average structure from the 25 NMR conformations. Relative solvent exposure was obtained using Biopython and DSSP [39]. Images of protein structure were rendered with the software Pymol [40].

#### Protein-protein docking

Each NMR conformer of PcFK1 was docked against the model of PfSUB1 using the ZDOCK software [41]. The docking search was limited to the binding region of PfSUB1 defined by the following residues:D34, H90, L123, K127, L128, G129, G151, S152, F153, S154, F155, D156, S179, A180, S181, N182, C183, K203, G266, T267, S268, M269. We performed the docking search using full rotational sampling and generated 2000 solutions per NMR conformer, for a total of 50.000 solutions. The best 2000 solutions were selected according to the distance between the P1 carbon-carbonyl and the hydroxyl of the catalytic serine of PfSUB1 (S268).

#### Docking refinement

We refined the complexes using a protocol of restrained molecular dynamics where the distance between the hydroxyl of the reactive serine of PfSUB1 was restrained between 3 to 5 Angstroms to the peptidic carbon in P1. We previously validated this procedure in a complex between a subtilisin and a protein inhibitor showing an improvement of the quality of the docking (Bastianelli *et al.* 2010, submitted). The refinement stage consisted of three phases, each one consisting of 120.000 steps. In the first phase, the receptor (PfSUB1) was kept “rigid” by applying a Cartesian restraint with a force-constant of 10 kcal/mol/Å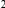, and the distance restraints were switched on/off every 15.000 steps. In the second phase a Cartesian restraint with a force-constant of 10 kcal/mol/Å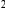 was applied only to heavy main-chain atoms with fully flexible side-chains, and in the third phase the force-constant of this Cartesian restraint was reduced to 0.01 kcal/mol/angstrom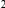. During these phases, the ligand (PcFK1) was kept completely flexible. An additional 1.0 ns of regular MD simulations was performed to allow the system to relax into its final configuration.

#### Molecular dynamic simulations

All molecular dynamics simulations (MDs) were performed using the SANDER code from the AMBER 9 [38] simulation suite and the ff99SB force-field [42]. Starting structures were first minimized in vacuum, and the system was then hydrated using TIP3P [43] water molecules and neutralized by adding an appropriate number of monovalent counterions. To remove bad contacts between solute and solvent molecules, we performed a two stage minimization protocol, using a Cartesian restraint on the solute with a force-constant of 10 kcal/mol/Å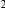 in stage 1, and 1 kcal/mol/Å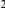 in the second for a total of 500 steps. During the equilibration/heating and production dynamics all covalent bonds to hydrogens were constrained with the SHAKE [44] algorithm. All simulations were performed under periodic boundary conditions with a time step of 2 femtoseconds. Electrostatic interactions were treated using the PME [45] (Particle Mesh Ewald) method with a direct-space sum cut-off of 8 Å. Rapid initial thermal equilibration to 300

K was performed over 20 ps under constant-volume conditions followed by 10 ps equilibration at constant-pressure. The production run MD simulations were performed using using a weak-coupling thermostat with a time constant of 2.0 ps and a pressure relaxation time of 2.0 ps. Post MDs processing and analysis was performed with the ptraj utility of AMBER 9.

#### Free energy calculations

For our calculations and analysis we used 50 snapshots (every 20 ps) out of 1 ns MD simulation for each solution. Residues at the interface were selected with the neighbor search implemented in Biopython and a distance cutoff of 5 Å between the center of mass of two aminoacids. The tool MM-PBSA [18] in AMBER was used to calculate the free energy decomposition. We used the GBSA model proposed by Tsui and Case [46] with an external dielectric of 80 and internal dielectric of 1.0. For calculating the nonpolar contribution, the surface tension coefficient (SURFTEN) was set to 0.0072 and the surface offset (SURFOFF) to 0.0. The solvent accessible surface area (SASA) was calculated using the ICOSA method. The entropy was estimated using the nmode tool in AMBER using a distance-dependent dielectric constant of 4 and a DRMS (convergence criterion for the energy gradient) of 0.0001.

### PcFK1 synthesis, folding and purification

PcFK1 was chemically synthesized with an extra serine at the C-term. We made this choice to have a sequence closely similar to the NMR structure of PcFK1,which carries a homoserine lactone. As there is no difference in activity [12] between the wild-type PcFK1 with an amide at the C-term and the one carrying the homoserine lactone, it is likely that the C-term does not play a role in the activity. To be sure of this we also tested the wild-type PcFK1 with PfSUB1, with no major changes in the activity if compared to the one carrying an extra serine at the C-term. Fully reduced PcFK1 (desalted, 35%–60% pure as assessed by HPLC) was obtained from GenScript Corporation, Piscatway, NJ, USA. We dissolved the peptide (50 mg) in 75 mL of 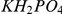 buffer (0.2 M, pH 8.2), allowing air-oxidation at room temperature under gentle stirring. Disulphide bridges formation was monitored with Ellman's test [47] and analytical HPLC (column ACE C18, 5 microns, 125×4.6 mm, eluent A: 0.1% TFA/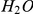, eluent B: 60% 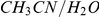/0.1% TFA) using a 30 min linear gradient of 25% to 55% B at 1 mL flow rate (monitoring at 210 nm). When Ellman
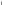
s tests were negative and HPLC monitoring showed no traces of starting materials (after 3 to 5 days), the reaction mixture was centrifuged and the supernatant loaded onto a preparative HPLC column (Merck Lichrospher 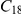, 10 microns, 250×25 mm). The peptide was eluted with a 90 min linear gradient of 25% to 55% B at 10 mL flow rate (monitoring at 220 nm). The fractions containing the oxidized peptide were combined and lyophilized to yield 15 to 30% of the desired peptide. Successful oxidation was confirmed by mass spectrometry (MALDI-Tof, Bruker Biflex III). All the experiments were performed using three different synthesis lots of PcFK1 and they all yielded similar results. The correct disulphide bond connectivity was confirmed by NMR spectra analysis.

### PfSUB1 and PvSUB1

#### Production

Recombinant baculoviruses expressing recombinant forms of PvSUB1 and PfSUB1 (described in Bouillon et Giganti *et al.*, in preparation) were amplified by infecting 5×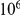 Sf9 cells in T-25 culture cultivated in Insect XPRESS medium (Lonza) supplemented with 5% fetal calf serum and 50 mg/L gentamycin. The final viral stock was titrated by end-point dilution assay. For large-scale protein production, Sf9 cells (1 L at 3×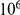 cells/mL) were infected for 72 h with recombinant baculovirus at a MOI of 10 in Insect XPRESS medium supplemented with 50 mg/L gentamycin and 0,5 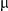
g/mL of tunicamycine.

#### Purification

Culture supernatant containing the secreted and active PvSUB1 or PfSUB1 recombinant enzymes was harvested, centrifuged 30 min at 2150 rcf to remove cells and cellular debris and concentrated/diafiltrated against D-PBS 0,5 M NaCl; 5 mM Imidazole (loading buffer). The protein was purified on an AKTA purifier system (GE Healthcare). The sample was loaded onto a 3 mL TALON Metal affinity resin (Clontech Laboratories) previously equilibrated in loading buffer, thus allowing the binding of PvSUB1 or PfSUB1 recombinant proteins via the addition of a 6×-histidines tag in its C-terminal. The column was extensively washed with loading buffer and the bound protein was eluted with a linear gradient from 5 to 200 mM imidazole in D-PBS 0,5 M NaCl. Fractions containing PvSUB1 or PfSUB1 were pooled concentrated using a Amicon Ultra 15 (10000 MWCO) and size fractionated onto a HiLoad 16/60 Superdex 75 column equilibrated with 20 mM Tris pH 7,5, 100 mM NaCl to remove imidazole and exchange buffer. Throughout the purification procedure, fractions were monitored by absorbance (280 nm) and analyzed by Coomassie blue staining of SDS-PAGE gels and activity assay. Fractions containing the PvSUB1 or PfSUB1 purified proteins were pooled and protein concentration was determined using the BCA Protein Assay following manufacturers recommendations (Bio Basic). Purified PvSUB1 or PfSUB1 recombinant proteins were stored at −20

C following the addition of 30% v/v of pure Glycerol.

### Enzymatic Test

For the kinetic assays we used the recombinant PvSUB1/PfSUB1 enzymes and specific peptide substrates whose sequence are deduced from the auto-maturation site of each one: KLVGADDVSLA, with cleavage occurs between the two aspartates for PvSUB1 and KLVSADNIDIS with is cleaved between the aspartate and the asparagine for PfSUB1. The substrates used had the fluorophores/quencher Dabsyl/Edans at each edge. The enzymatic assay were performed with 13 ng of purified PvSUB1 or PfSUB1 in 20 mM Tris pH 7.5 and 25 mM CaCl2 at 37

C. The apparent K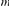 of PfSUB1 and PvSUB1 for their substrate being 30,2 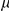
M
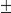
3,4 and 19,7
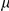
M
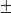
1,7 respectively (described in Bouillon et Giganti et al, in preparation), all further experiments were performed using 25 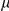
M of substrates. For the determination of the K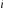, the compounds, previously resuspended in ultra-pure distilled water at 10 mM, were tested at ten different concentrations ranging from 1 mM to 2 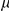
M following sequential 1∶2 dilutions. The final mixture was distributed in duplicate into a 384-well black microtiter plate (Thermo Scientific) and the fluorescence was monitored every 3 minutes for 90 min at 37

C in a Labsystems Fluoroskan Ascent spectro fluorometer using the excitation and emission wavelengths of 460/500 nm. The slope of the linear part of the kinetic was determined in an Excel (Microsoft) spreadsheet. Every steps of the enzymatic assay were done on ice to make sure that the protein was not active before the measure of the fluorescence. The K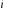 and IC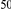 values were determined (N = 3) using GraphPad Prism software.

### Culture Tests

Asexual cultures of reference clone 3D7 obtained from MR4 (www.MR4.org) was maintained in continuous cultures following the method of Trager an Jensen [48], except that the medium was composed of RPMI 1640 medium supplemented with 10% decomplemented human serum (AB+), hypoxanthine 100 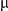
M, gentamycin 50 ng/mL. Parasites were incubated at 37

C in an atmosphere composed of 5%O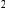, 5%CO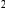 and 90%N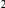. Quantitative assessment of the antimalarial activity of PcFK1 was performed as described by Desjardins *et al.* [49] and Bougdour *et al.* [50] on asynchronous culture of clone 3D7 (0,5% parasitemia and 1% hematocrit), except that the parasites were in contact with the drug for 48 hours, the culture medium contained 10 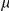
M hypoxanthine. EC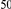 have been determined following nonlinear regression analysis using HN-NonLin V1.1 software (http://malaria.farch.net).

A synchronised culture composed of segmented schizontes of *P. falciparum* (3D7 clone) at 0.5% parasitemia and 1% hematocrit is performed in a 24 wells plate. An aliquot corresponding to 10% of the starting culture (T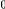) is diluted in a solution at 0.04% of glutaraldehyde in PBS (Dulbecco) and store at 4

C for further flow cytometry analysis. E64 (trans-Epoxysuccinyl-L-leucylamido(4-guanidino)butane, Sigma-Aldrich), a cysteine-protease inhibitor known to block the egress of *P. falciparum* merozoites *in vitro* [51] was used as a positive control at a final concentration of 10 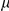
M, while PcFK1 was tested at a final concentration of 200 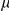
M and a mock control (T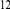) received sterile water, in which PcFK1 was resuspended. The final experiment ended after an incubation of 12 hours, allowing the rupture of the parasitized erythrocytes, the egress of merozoites and their subsequent entry into fresh red blood cells. 10% of the cultures are resuspended in a solution of 0.04% glutaraldehyde in PBS (Dulbecco) and store at 4

C for flow cytometry analysis. The progress of the parasitaemia from segmented schizont to newly formed trophozoites was assessed by flow cytometry after staining samples by the DNA-binding fluorescent dye, YOYO-1, as previously described by Li and colleagues [52] with some modifications. Briefly, following a centrifugation at 450 g for 5 min the pelleted cells were re-suspended in 0.3 ml PBS (Dulbecco) supplemented with 0.25% Triton X-100 and incubated for 10 min at room temperature. After centrifugation, the permeabilized cells were re-suspended in 500 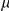
L of RNase at 50 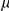
g/mL and incubated for at least 3 h at 37

C. Then YOYO-1 solution (Invitrogen) was added to obtain a final concentration of 500 ng/mL. Samples were incubated at 4

C in darkness for at least 4 h and centrifuged at 450 g for 5 min. The pelleted cells were re-suspended in 0.3 ml PBS before being analysed by flow cytometry using a FACSCanto(BD) apparatus and the data were analyzed using FlowJo (Tree Star) software. The fluorescent signal of YOYO-1 dying cells was collected in FL-1 channel after compensation of fluorescent intensity in the FL-2 channel.
